# Tailored approach to the choice of long-term vascular access in breast cancer patients

**DOI:** 10.1371/journal.pone.0255004

**Published:** 2021-07-22

**Authors:** Hyangkyoung Kim, Sukyung Kwon, Soo Mi Son, Eunseon Jeong, Jang-Yong Kim

**Affiliations:** 1 Division of Vascular Surgery, Department of Surgery, College of Medicine, University of Ulsan and Asan Medical Center, Seoul, Korea; 2 Division of Vascular Surgery, Department of Surgery, College of Medicine, Pusan National University Yangsan Hospital, Yangsan, Korea; 3 Division of Vascular and Transplantation Surgery, Department of Surgery, College of Medicine, Seoul St. Mary’s Hospital, The Catholic University of Korea, Seoul, Korea; Ohio State University Wexner Medical Center Department of Surgery, UNITED STATES

## Abstract

This study compared the possible options for vascular access in breast cancer patients by analyzing the complications of each method. We retrospectively evaluated the vascular access procedures for intravenous chemotherapy in breast cancer patients from 2016 to 2018. A total of 300 consecutive patients were included, 100 each who received peripherally inserted central catheters (PICCs), arm ports, and chest ports. When selecting a catheter, a PICC was considered when four cycles of chemotherapy were expected. Otherwise, patient preference was considered. All but one patient with an arm port were women, with mean age of 51.7 ± 9.1 years. The total mean complication-free catheter indwelling time was 1357.6 days for chest ports, 997.8 days for arm ports, and 366.8 days for PICCs (p = 0.004). There were 11 catheter-related complications (3.7%), one in a chest port patient, five in arm port patients, and eight in PICC patients. There was no patient with catheter related blood stream infection or deep vein thrombosis. All three types of catheters could be used in breast cancer patients without causing serious complications. The selection of catheter considering the clinical situation was effective for providing a safe and secure chemotherapy delivery route.

## Introduction

In patients with an advanced stage of breast cancer, adjuvant intravenous chemotherapy is often required to improve survival. Despite the well-recognized necessity for safe and secure venous access for intravenous chemotherapy, the choice of vascular access for breast cancer therapy varies in practice and there is a lack of consensus regarding the best choices. The type of vascular access, duration of treatment, and type of chemotherapeutic regimen as well as the vessel condition of the patients are factors affecting the suitability of each option [1]. The currently available vascular access devices are a traditional peripheral intravenous line or central vascular access such as a peripherally inserted central catheter (PICC) or a surgically inserted central line to the chest (chest port) or the arm (arm port). Each catheter option is associated with advantages and drawbacks. The perceived benefits of each type of access must be balanced against the procedure-related risks and complications such as infection and thrombosis and against the costs and time for insertion and ongoing maintenance [2]. To date, no study has compared the three options for central venous accesses in breast cancer patients. Given the variability in the potential vascular access choices and clinical situations of the patients, this study was conducted to compare the catheter indwelling time and the complications of each access method in breast cancer patients.

## Materials and methods

We retrospectively evaluated breast cancer patients who underwent vascular access procedures for intravenous chemotherapy in Seoul St. Mary’s Hospital of the Catholic University of Korea from June 2016 to June 2018. Patients older than 18 years old with a life expectancy longer than six months and requiring chemotherapy through a central venous catheter were eligible for inclusion in the study. Patients with ongoing severe systemic infections, clinically significant upper extremity/central deep vein thrombosis (DVT), severe coagulopathy, the inability to communicate, or an imminent need for a dialysis fistula were excluded from the study. A total of 300 consecutive patients, 100 each with PICCs, arm ports, and chest ports were included in the analysis. The study was approved by the Institutional Review Board (IRB) of the Catholic Medical Center at the Catholic University of Korea (KC18OESI0728) and complied with the Declaration of Helsinki. The requirement for informed consent was waived. Our research team accessed the database on June 19, 2019.

**Fig 1:**
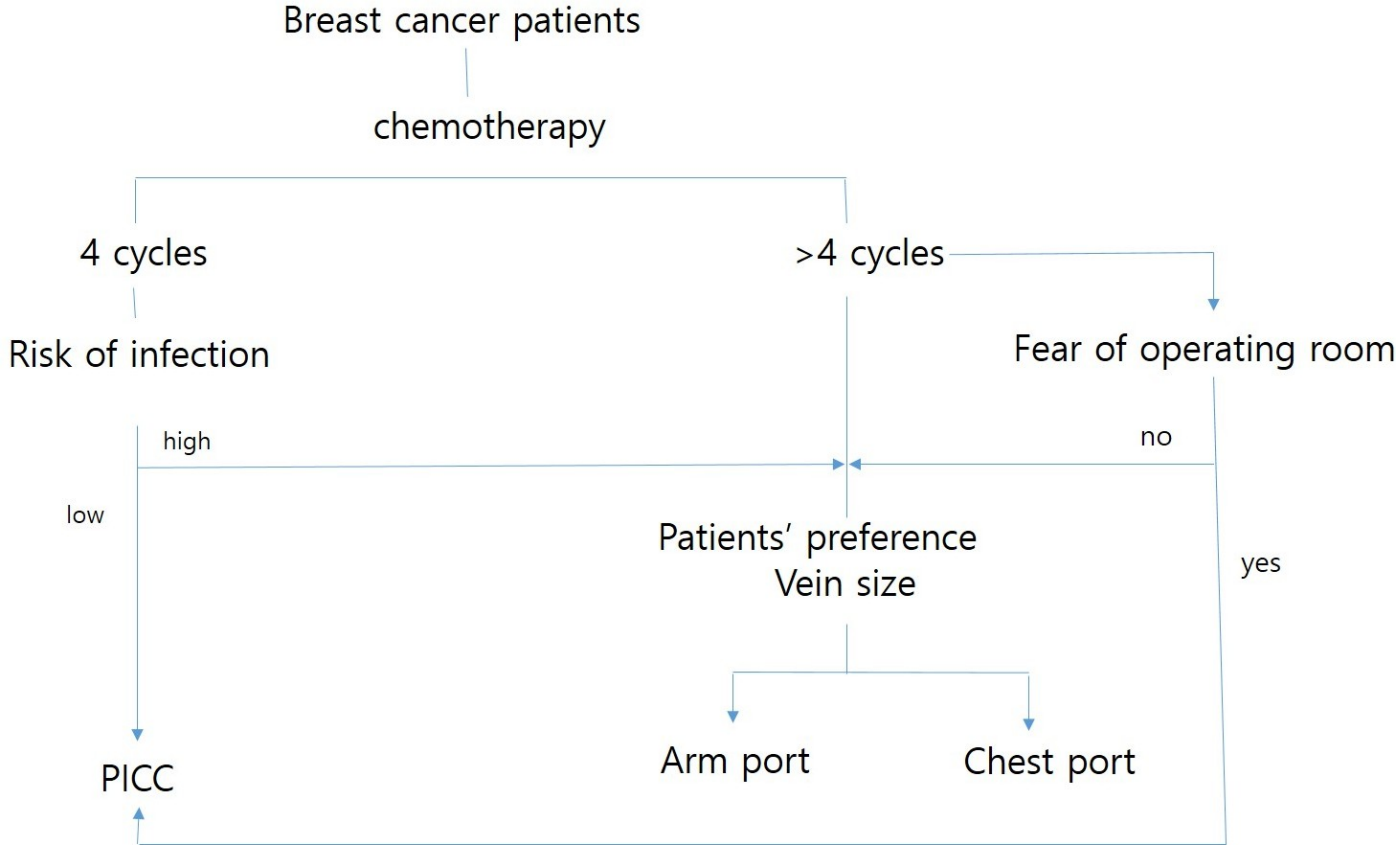
Decision algorithm to select the proper vascular access.

The appropriate chemotherapeutic program, such as treatment with cyclophosphamide, methotrexate, 5-fluorouracil, doxorubicin, trastuzumab, epirubicin, paclitaxel, or Taxotere, was adopted based on the patient’s condition. The type of catheter was selected by the physician’s decision or the patient’s preference as shown in Fig 1. Since the activity of the upper extremity of the patient is restricted after PICC catheterization, we preferably inserted totally implanted vascular access devices (TIVADs) when more than six chemotherapy cycles were expected. If the patient was at an increased risk of infection from their daily activities or preferred a totally implanted device, TIVAD was selected. The chest or arm location of the infusion reservoir was decided according to the patient’s preference. If the patient was reluctant to go to the operating room for insertion and removal, PICC was selected.

The primary outcome was clinically significant catheter-associated complications including DVT, infections, and skin problems. A catheter-associated DVT was defined as a DVT related to relevant signs or symptoms (pain, redness, swelling, or tenderness) with confirmation by ultrasound, and/or computed tomography (CT) or a DVT incidentally found on imaging for other purposes. No periodic imaging was conducted to identify silent thrombosis. Catheter-associated infections were defined according to the Infectious Diseases Society of America criteria [3]. The secondary outcomes were any events requiring unplanned catheter removal or change.

### Catheterization method

PICCs were inserted at the outpatient clinic and TIVADs in the operating room. The materials used were the Groshong PICC 4Fr (BD, Bard Access Systems, Inc., Salt Lake City, UT, USA) for the PICCs, titanium Vital-Port 5 or 6Fr (Cook Medical, LLC, Bloomington, IN, USA) for the arm ports and the Power Port 9.6Fr (BD, Bard Access Systems, Inc.) and the titanium Vital-Port 6 or 8Fr (Cook Medical, LLC) for the chest ports. The position of the catheter tip was targeted to the junction between the superior vena cava and the right atrium. X-rays were taken to confirm the tip position after catheter placement.

#### Chest port implantation

The contralateral side of the breast cancer was selected for chest port implantation to avoid the radiation field. Chest port implantation was usually performed through the internal jugular vein (IJV). After local anesthetization with 1% lidocaine, the IJV was punctured under direct ultrasound visualization with a micropuncture set, then a 0.035” guidewire was introduced through the set. After the catheter was placed, the subcutaneous tunnel was made. A subcutaneous pocket for the infusion chamber was made 0.5–1.0cm below the skin. The catheter was connected to the infusion device, and then the catheter was inserted through a peel-away sheath under fluoroscopic guidance. The tip of the catheter was positioned below the right bronchus. A noninvasive Huber needle was inserted into the bottom of the port vertically through the skin to check the patency of the catheter. The wound was closed with 4–0 absorbable sutures.

#### PICC

The puncture site was 2-finger widths above the elbow. The preferred needle insertion vein was the basilic vein, followed by the cephalic vein, and the brachial vein. A tourniquet was tightened around the upper arm, and skin preparation and draping were performed. The venous puncture was done with a 21-gauge micropuncture needle after the application of a topical anesthetic. When performed under ultrasound guidance, a 12 MHz linear array probe was used, which was draped with a sterile plastic sleeve. A micropuncture guidewire was introduced into the vein through the needle and advanced blindly after confirmation of its location within the vein by ultrasound and release of the tourniquet. The needle was withdrawn over the wire and a peel-away sheath introducer was advanced over the guidewire into the vein. Following the removal of the dilator, the wire was removed before the catheter was introduced through the sheath to the desired position. The sheath was peeled away and placement was completed after checking if the catheter was in the IJV using ultrasound. Then, the catheter was covered and fixed with a sterile transparent film.

#### Arm port

The venous puncture was performed in the same manner as for the PICC in the contralateral arm. A subcutaneous pocket and tunnel were made near the puncture site in the same manner as for the chest port. The catheter was inserted through the peel-away sheath under fluoroscopic guidance. The tip of the catheter was positioned below the right bronchus. Then, the catheter was connected to the infusion device. After checking the patency of the catheter with the Huber needle, the wound was closed with 4–0 absorbable sutures.

### Statistical analysis

The categorical data were analyzed by the Chi-squared test, and the continuous data were analyzed by ANOVA or the Kruskal-Wallis test for comparisons between the groups after the normality tests. Post hoc analysis was conducted using Tukey’s b method. The analysis of adverse event-free catheter survival was performed using the Kaplan-Meier method and the log-rank test. Cox regression model was constructed to evaluate the independent predictors of catheter-related adverse events including thrombosis, occlusion, and infections during follow-up. All P-values were two-tailed, and a P-value of < 0.05 was considered statistically significant. Statistical analyses were performed using SPSS version 21 (IBM, Armonk, NY, USA) and R software version 4.0.2 (R Development Core Team, 2006).

## Results

The demographic data are summarized in Table 1. All but one patient with an arm port were women, with ages ranging from 28 to 78 years (mean age 51.7 ± 9.1 years, median age 51 years). The mean body mass index (BMI) was 23.3 ± 3.2. The mean age and BMI were not significantly different between the groups. All catheters were inserted in the contralateral side, except for in three patients with bilateral cancer. In the three patients with bilateral cancer, one patient’s catheter was inserted into an arm port on the right side and two patients had chest ports inserted on the left side. All chest ports were inserted through the IJV. The arm ports were inserted through the basilic vein in 70 patients, the cephalic vein in 23, and the brachial vein in seven patients. PICCs were inserted through the basilic vein in 84 patients and the brachial vein in 16. The mean (median) number of chemotherapy cycles was 5.96 ± 2.6 (6), six for patients with chest ports, 7.3 ± 2.9 (8) for patients with arm ports, and 4.82 ± 1.6 (4) in PICC patients (p < 0.01).

**Table 1:** Baseline demographic data.

	Chest port	Arm port	PICC	p-value
**Mean age**	51.7±9.4	51.9±8.8	51.3±9.1	>0.05
**Body mass index**	23.4±3.1	23.1±3.6	23.4±2.9	>0.05
**Cancer**				
**Cancer location**				>0.05
Right / Left	48 / 50	47 / 52	48 / 52	
Bilateral	2	1	0	
**Cancer stage**				
0	0	2	0	
IA / IB	22 / 0	13 / 4	45 / 2	
IIA / IIB	34 / 22	34 / 22	27 / 9	
IIIA / IIIB / IIIC	14 / 3 / 4	8 / 5 / 7	9 / 0 / 5	
IV	1	5	3	
**Operation**				
No operation	6	4	2	
Wide excision/quadrantectomy	51	61	69	
Mastectomy	41	34	27	
Lymph node dissection only	2	1	2	
**Lymph node dissection**				0.01
Sentinel node procedure	46	49	72	
Axillary lymph node dissection	47	46	21	
**Chemotherapy**				
Number of cycles	6 (4–8)	8 (6–8)	4 (4–5.75)	<0.001
Neoadjuvant chemotherapy	71	73	91	0.003
**Puncture site**				>0.05
Internal jugular vein	100	0	0	
Basilic vein	0	70	84	
Cephalic vein	0	23	0	
Brachial vein	0	7	16	
Indwelling duration (days (IQR*))	193 (134–312.5)	187.5 (168.5–225)	104 (65–151)	<0.001
**Catheter size**				<0.001
4 Fr	0	0	100	
5 Fr	0	47	0	
6 Fr	3	53	0	
8 Fr	22	0	0	
9.6 Fr	75	0	0	

*IQR, interquartile range.

There were no technical failures. There were 10 deaths during the follow-up period, two were patients with chest ports and eight were patients with PICCs. The causes of death were associated with the progression of cancer. There were eight events (2.7%) requiring unplanned catheter removal or changes, two in patients with chest ports, and six in patients with PICCs. there were no unplanned changes in the arm port patients. One patient developed peripheral neuropathy after PICC placement and it was removed on the day of insertion. Five patients changed to chest ports during usage due to contact dermatitis in two and changes in the chemotherapeutic regimen in three patients. Chest ports were removed due to the refusal of further treatment in one patient and a wound infection in one patient.

There were 11 catheter-related complications or changes where the catheter was not used as planned (3.7%, Table 2), one in a patient with a chest port, five in patients with arm ports, and eight in PICC patients. The chest port-related complication was a wound infection (n = 1). No pneumothorax or hemothorax occurred. The arm port-related complications were an infusion chamber dislocation (n = 1), catheter-related thrombosis (n = 3), and an elbow contracture (n = 1). In the three catheter-related thrombosis patients, the insertion site was the basilic vein in one patient and the cephalic vein in two patients, and all had 6 Fr catheters. Venous thrombosis was confined to the superficial veins in three patients. The PICC-related complications were neuropathy (n = 1), inadvertent pulling-out (n = 1), and dermatitis (n = 3). No patients developed catheter-related bloodstream infections (CRBSIs). There was no statistically significant difference in the thrombosis rate between the patients with PICCs and those with arm ports (p > 0.05). The complications were not significantly different between the catheter types (p = 0.214). The factors related to the complications were analyzed and are summarized in Table 3. No factor was significantly associated with the complications (all, p > 0.05). Complication-free rates were not significantly different among the groups at the 3-month follow-up (99.0% for chest port, 97.0% for arm port, and 96.2% for PICC; p = 0.509) or 6-month follow-up (99.0% for chest port, 94.6% for arm port, and 92.2% for PICC; p = 0.121).

**Table 2:** Complications.

	Chest port	Arm port	PICC	p-value
**Venous thrombosis**	0	3	0	0.036
**Wound infection**	1	0	0	>0.999
**Elbow contracture**	0	1	0	>0.999
**Contact dermatitis**	0	0	3	0.036
**Neuropathy**	0	0	1	>0.999
**Catheter position change**	0	1	1	0.777
**Total**	1	5	5	0.214

**Table 3:** Factors associated with catheter-related complications.

	Unadjusted OR						
	Coeff.	S.E	Wald	p-value	OR	95% CI of OR	
						Lower	Upper
**Age**	-0.003	0.033	0.008	0.928	0.997	0.934	1.064
**Body mass index**	0.003	0.093	0.001	0.974	1.003	0.836	1.204
Catheter (PICC)*	0.936	0.625	2.241	0.134	2.549	0.749	8.676
Catheter (Arm port)*	0.311	0.609	0.261	0.609	1.365	0.414	4.502
**Catheter size**	-0.293	0.181	2.622	0.105	0.746	0.523	1.064
**Catheter duration**	-0.001	0.002	0.335	0.563	0.999	0.995	1.003
**Number of chemotherapy cycles**	-0.116	0.118	0.973	0.324	0.890	0.707	1.121
**Catheter laterality (Left)**	-0.068	0.606	0.012	0.911	0.935	0.285	3.064
**Catheter location (arm)**⁋	1.756	1.050	2.798	0.094	5.789	0.740	45.314

*compared to the chest port.

⁋compared to the chest.

coeff, coefficient; CI, confidence interval; S.E., standard error; OR, odds ratio; PICC, peripherally inserted central venous catheter.

**Fig 2:**
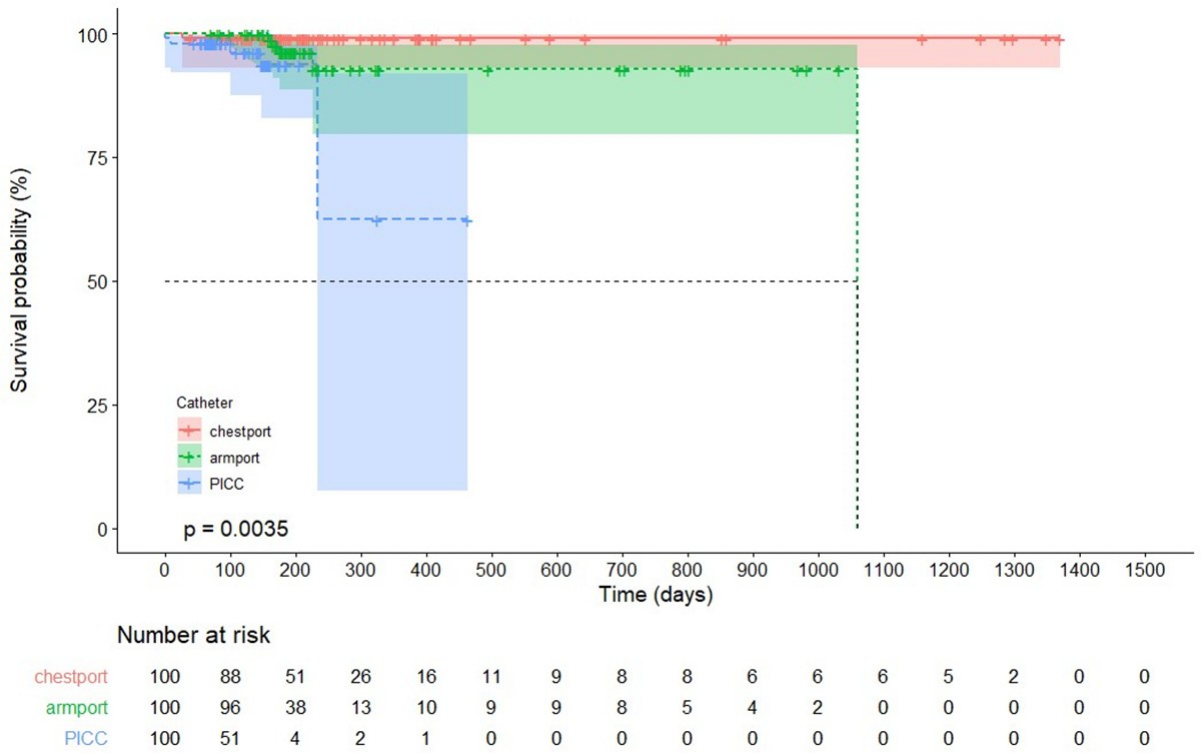
Cumulative incidence of complication-free catheter usage rate.

The median catheter indwelling duration was 193 days (interquartile range (IQR) 134–312.5) for patients with chest ports, 187.5 days (IQR 168.5–225) for arm ports, and 104 days (IQR 65–151) PICCs. The total complication-free mean catheter indwelling time was 1253.2 days (95% CI: 1167.0–1339.3), 1357.6 days (95% CI: 1331.3–1383.8) for chest ports, 997.8 days (95% CI: 924.0–1071.6) for arm ports, and 366.8 days (95% CI: 250.9–482.7) for PICCs (Fig 2, p = 0.004). There was no significant difference between the ports (p = 0.16).

## Discussion

In the treatment of breast cancer, long-term chemotherapeutic regimens are commonly required [4]. Secure venous access is necessary for these treatments as chemotherapy drugs act as vesicants to the veins [5]. Currently, the most preferred options for long-term chemotherapy are central vascular access devices including TIVADs, such as chest or arm ports, and PICCs. These indwelling vascular accesses enable the delivery of chemotherapeutic drugs or blood products and can be used for blood draws, avoiding the possible local complications associated with the extravasation of cytotoxic drugs [6, 7]. Each approach has different safety and risk profiles as well as effects on the patient’s quality of life [8, 9]. The rate of major complications from TIVADs including both chest and arm ports ranged from 3.5 to 19% in previous studies [10–12]. TIVADs may be associated with complications such as infection, thrombosis, migration or malposition, pain associated with the procedure, and catheter occlusion [1, 13, 14]. The common complications of PICCs are infection, thrombosis, catheter occlusion, migration, tip malposition, rupture, and phlebitis [15, 16]. The complication rates of PICCs were reported to range from 4 to 50% [13, 17–20], which is higher than the rates associated with ports. However, PICCs have the advantage of being peripherally placed at the bedside and are easier to insert and remove than TIVADs. Despite being an imperative decision in clinical practice, information on the relative risks, benefits, and costs of the various methods of venous access, especially for breast cancer patients, is still lacking.

In our study, the clinicians had a preferred type of catheter depending upon the specific clinical situation and we investigated the feasibility of this approach. In this tailored approach, none of the three types of catheters were associated with serious complications including deep vein thrombosis or CRBSIs. Particularly breast cancer patients in whom axillary dissection has been performed have an increased risk of lymphedema and possible cellulitis after arm catheterization. It is important to preserve venous flow and prevent lymphedema in patients with breast cancer after insertion of the port via the ipsilateral arm or subclavian vein [21, 22]. In addition, TIVADs on the ipsilateral chest or neck are best avoided in patients with breast, lung, or head and neck malignancy requiring radiation therapy [22]. In our series, PICCs or arm ports could be a safe option when the contralateral arm was selected, especially in patients who underwent axillary lymph node dissections. In bilateral breast cancer patients without axillary dissection, a chest port on either side or an arm port were possible options and there were no complications in those patients. With this approach, the complication rate of chest ports was 1% (1/100) even with the longest indwelling time. Moreover, there were no severe infection complications in any patients, which seemed to be related to the characteristics of the breast cancer patients of relatively young age and female sex that distinguish them from other patients with other types of cancers.

The major finding in this study was that the overall rate of adverse events in the PICC group was comparable to that of the chest or arm port groups, and most of them were local complications at the insertion site such as contact dermatitis. Consideration should be given to the potential risk factors for central venous access device-associated skin impairment when choosing a PICC, and there seems to be room for improvement in the rate of these complications. Chest ports showed the most excellent results in terms of complication rates and catheter indwelling time. A previous study suggested that peripheral arm ports were comparable to chest ports in arterial injuries and had the prominent advantage of less pneumothorax [23]. Since we used ultrasound guidance in all cases, we did not encounter arterial injury or pneumothorax. This practice seemed to yield excellent results for chest ports, particularly in our study. Catheter-related thrombosis developed only in arm port patients in this study, in contrast to previous studies that reported that PICCs were associated with higher DVT rates [16, 24, 25]. The main differences between the PICC group and the arm port group in our study were the vein utilized for catheterization and the catheter indwelling time. It was difficult to compare the catheter indwelling time of PICCs and arm ports because PICCs are intended for use in patients who will receive short-term chemotherapy. The indwelling time in in the three patients with catheter-related thrombosis were longer than the mean PICC indwelling time. Thus, catheter duration might serve as one of the major risk factors for thrombosis. Moreover, all three patients with catheter-related thrombosis had 6Fr catheters, whereas all the patients in the PICC group had 4Fr catheters. The result is consistent with previous literature that the large catheter size is a risk factor for DVT [26]. In the case of long-term chemotherapy patients whose catheter size is relatively large compared to the vein size, it is considered to be a better option to consider the chest ports.

This study investigated the feasibility of all possible CVC options in breast cancer patients who are receiving long-term chemotherapy. We tried to generate a catheter selection algorithm for the most desirable catheter. In addition, we incorporated the patient’s preference and fear of the operating room into the catheter decision. Our study had obvious limitations from its retrospective and non-randomized design. However, the catheter selection could not be made uniform because the choice was based upon diverse clinical situations.

## Conclusion

All three types of catheters could be used in breast cancer without causing serious complications. The tailored approach to selecting the appropriate type of venous access for certain clinical situations was effective for providing a safe and secure chemotherapeutic route.

## Supporting information

S1 Data Data. (XLSX)
